# Impact of the COVID-19 Pandemic on China's Stock Market Volatility, During and After the Outbreak: Evidence From an ARDL Approach

**DOI:** 10.3389/fpubh.2022.810102

**Published:** 2022-05-18

**Authors:** Cheng Jin

**Affiliations:** School of Business, Shaoxing University, Shaoxing, China

**Keywords:** COVID-19, ARDL bounds test, IBOLR, policy withdraw, stock volatility

## Abstract

**Purpose:**

In this study, we empirically investigate the impact of the COVID-19 pandemic on China's stock price volatility during and after its initial outbreak, using time-series daily data covering the period from July to October, 2020 and 2021, respectively.

**Design/Methodology/Approach:**

In the estimation, the ARDL bounds test approach was employed to examine the existence of co-integration and the relationship of long-run and short-run between the new infection rates and stock price volatility, as stable and unstable variables are mixed. The inner-day and inter-day volatility, based on the Shanghai (securities) composite index, are estimated in separate empirical models. In addition, the Inter-bank overnight lending rate (IBOLR) is controlled in order to consider the effect of liquidity and investment cost.

**Findings and Implications:**

We find that in the initial year (2020) of the epidemic, the new infection rate is negatively correlated to stock prices in the short-term, whereas no significant evidence existed in the long-term, regardless of model specifications. However, after the epidemic's outbreak (2021), the result depicts that new infections increased stock prices in the long-term, and depressed its inner-day volatility in the short-term, which is inconsistent with most investigations. This phenomenon may be due to the fact that investors were more concerned about the withdrawal of monetary easing and fiscal stimulus, which were introduced to fight against the epidemic's impact on economy, than the epidemic itself. This study complements the limitations of most existing studies, which just focus on the period of the epidemic's outbreak, and provides insight into macroeconomic policy making in the era of the post COVID-19 epidemic such as the structural and ordered exit of the stimulating policies, intervention in IBOLR and balance social and economic sustainability.

## Introduction

As the most influential health crisis of the recent decade, the COVID-19 pandemic has triggered tremendous social and economic influences among countries in the past 2 years. There is a proliferation of papers investigating its sudden impact on the stock market with most of their perspectives suggesting that the impact is devastating (1–3). Despite the increase or decrease in prices, the volatility itself is also of great significance to the market (1, 4, 5). China's stock market is the world's second largest, behind the US,^1^ and is inextricably linked to economic development and social stability, its yields have a global spillover effect (6). Moreover, due to current mass vaccination, proficiency in countermeasures and adaptation of investors' psychology, it is possible to yield different impacts on the stock market to the extensive literatures which mainly focus on the period of its initial outbreak. Studying the correlation of COVID-19 and China's stock market, during and after the outbreak, is helpful in detecting the pandemic's potential external shock on capital markets and shed light on policy implications to promote a smooth transition and balanced development in society and the economy to achieve sustainable development goals (SDG) in the forthcoming years. The light can even contribute to investors making risk management and portfolio assets allocation in the stock market (7).

The aims of the study are to examine the long- and short-term impacts of the COVID-19 pandemic on China's stock market volatility during and after its outbreak, detect the differences in their influences and provide possible tailored policy suggestions. The time range covers July to October (2020 and 2021, respectively) for the reason that in these periods, during both of the 2 years the macroeconomic variables are relatively stable, whereas in other periods the fiscal and monetary policies, international supply chains, main trade partners' epidemic and commodity prices experienced drastic changes. ADRL modeling has advantages in dealing with variables integrated with different orders (8, 9) and small samples (10). Compared to other techniques, the ARDL method can also provide long and short-term coefficients simultaneously, with an error correction model. In our study, we firstly make a unite root test to detect if the variables' are stationary or unstationary. Its result suggests that the dependent variables and independent variables are combined with *I(0)* and *I(1)*. Therefore, we employ the ARDL approach to investigate the existence of co-integration and both the long and short-term effects of the epidemic on China's stock market. In the estimation, we consider three aspects of volatility: the increase rate, the inner-day and inter-day volatility, using separate empirical models. Confirmed cases and death cases are usually used as influencing factors (3, 11, 12). Since 2021, the epidemic in China has been fading out with almost no death cases, we only consider the data of confirmed cases per million people as a unifying measurement. The inter-bank overnight lending rate is considered in order to reflect on the impact of market liquidity and investment cost on the stock index.

The remaining part of the paper is organized as follows. Section Literature Review: presents the theoretical background and related literature. Section Data and Empirical Model: describes data descriptions, empirical equations, and estimation methods. Section Empirical Results: provides a series of empirical results. Section Conclusions and Policy Implications: contains some conclusions and policy implications.

## Literature Review

Public health emergencies have been suggested to hit global economic growth, social stability and cause upheaval in financial markets (5, 13–15), for instance, SARS in 2004 (16), H1N1 in 2009 (17), and EBOV in 2014 (18). Currently, the COVID-19 pandemic is also regarded as exerting significant influence on the economy (12, 19, 20), even surpassing that of the previous health crisis (21). Specifically, the epidemic is attributed as disrupting production, distribution and the supply chain (22), in changing personal consumption patterns (23, 24), investors' behaviors (25), and business confidence (23), therefore impacting the entire economy and channels to the financial market. Besides downside risks in financial or stock markets, it's denoted to increase fear-induced sentiment (26–28) and resulting economic uncertainty (29). Economic uncertainty may also be a result of government reaction triggered by the pandemic (30), such as prohibiting human mobility and manufacturing lockout (12, 31). Kalyvas et al. (29) and Sharif et al. (32) suggest that this uncertainty leads to a financial crash risk and to stock markets tumbling. In addition, risks are suggested to be touched off by a possible “domino” effect. Some institutions' or sectors' problems may spill over to endanger the entire market (33–37).

Numerous studies have specifically investigated the epidemic's assaults on the stock market. Based on the US, UK, Japan, and other developed countries' evidence, Bai et al. (2) and Rahman et al. (3) argue a significant negative influence of COVID-19 on stock prices and a positive influence on stock volatility. The adverse impacts of the epidemic on stock price or fluctuations are also addressed in emerging markets (1, 4, 28). Particularly, some of the investigations reveal that the reaction of the stock market is in the short-term (38, 39). Additionally, the unpredictability of the pandemic process is suggested to raise volatility (40). To put it another way, less unpredictability in the epidemic process tends to reduce stock volatility. It is consistent with risk aversion in portfolio investment suggested by Modern Portfolio Theory (MPT) and Capital Asset Pricing Model (CAPM), which use the Standard Deviation (*SD*) as the volatility to detect the relationship between expected return and investment risk. There is even attempt to explore the cross-region spillovers between countries' stock markets hit by COVID-19 (15). Nevertheless, few literatures focus on the influence of COVID-19 in the post-epidemic period, let alone their comparison, which may currently yield more practical significance.

Longstanding and extensive studies have suggested that there is an important relationship between interest rates and stock prices. Nozar and Philip's (41) investigation reveals that the immediate response of US stock prices is significant and dynamic, similar conclusions can also be seen in studies such as Fama (42), Rahman et al., (3) and Pal and Mittal (43). Specifically, in Germany and the UK, the interest rate's shock even accounts for approximately half of stock price's movement (44). However, the direction of the interest rates impacts proposed by prior studies is still ambiguous. For example, Amado (45) denotes that the effect of interest rates on stock prices is heterogeneous to industry characteristics and conditionally on the direction of interest rate change, whereas Hogan (46) and Alam and Uddin (47) address it as positive and negative, respectively. Besides, interest rates may affect a firm's profits, nest present values of future cash flow, and even stock transaction cost, in case a significant amount of stock purchasing is by borrowed money, therefore affecting stock value (48). In addition, although inflation and money supply are regarded as impacting stock prices based on the money demand theory and the dividend discount model (3, 49–54), they are indicated to finally increase or decrease the interest rate (55). Hence, in the investigation we employ the interest rate; here inter-bank overnight lending rate, as the control variable in the ARDL approach.

## Data and Empirical Model

The data sets utilized are daily new infection rate, stock price volatility, and the inter-bank overnight lending rate in China. We choose the data scope ranging from July to October (2020 and 2021, respectively) because in this period of 2 years the macroeconomic policy and international economic background are relatively stable. Hence, it is more suitable than any other period to detect the impact of the epidemic on the stock market and in order to make comparisons. Each day's COVID-19 new infection rate (henceforth CNIR) is measure by new infections per million people in China. Because the number of new infections is officially released every other day at 9:00 a.m., the independent variable of CNIR refers to the epidemic situation of the previous days. Likewise, the inter-bank overnight lending rate (henceforth IBOLR) is controlled in the estimation.

The stock price volatility consists of three aspects: the increase rate, inner-day volatility, and inter-day volatility, which are examined in separate models. Their calculating formulas are as follows:

(1) $$\begin{array}{r} INCR_{t}=\frac{index_{close\;t}\;-index_{close\;t-1}}{index_{close\;t-1}} \end{array}$$

(2) $$\begin{array}{r} INEDV_{t}=\frac{index_{close\;t}-index_{open\;t}}{index_{open\;t}} \end{array}$$

(3) $$\begin{array}{r} INTDV_{t}=\frac{index_{open\;t}-index_{close\;t-1}}{index_{close\;t-1}} \end{array}$$

Here, *INCR* represents the increase rate, measured by the growth rate of the closing index to the previous day's; *INEDV* is the inner-day volatility, which is the disparity rate of the closing index to the opening index; *INTDV* is the inter-day volatility, calculated by the change rate of the opening index to the previous day's closing index. Besides *index*_*open*_, *index*_*close*_ denotes the opening index and closing index of China's stock market, using Shanghai Securities Composite Index (SSCI). Subscript *t* in the variables signifies the day of the stock index, and Subscript (*t*-1) is the previous day of *t*. The data of SSCI are drawn from the website of the Shanghai Stock Exchange. In particular, the days that exist missing values are gotten rid of in the estimation. In order to unify the scale and make the value positive for further logarithm, the independent variables are normalized before estimation, as is shown in the following method:

(4) $$\begin{array}{r} fy_{t}=\frac{y_{t}-int\left(y_{\min}\right)}{y_{\max}-int\left(y_{\min}\right)} \end{array}$$

Here, y denotes the independent variables in the estimation, such as *INCR*, *INEDV*, and *INTDV*. *y*_min_ and *y*_max_ is the minimum value and maximum value of y. *int* is integral function, signifying the maximum integer that does not exceed the value of *y*_min_. The summary statistics of the variables is provided in Table 1.

**Table 1:** Summary statistics of main variables.

	**Mean**	**Median**	**Maximum**	**Minimum**	**Std. dev**.	**Observations**
CNIFa	0.05	0.02	0.16	0.01	0.04	50
INEDVa	0.53	0.52	1	0.09	0.22	50
INTDVa	0.64	0.64	1	0.18	0.13	50
INCRa	0.53	0.53	1	0.02	0.17	50
IBOLRa	1.87	1.94	2.40	0.68	0.35	50
CNIFb	0.036	0.03	0.09	0.01	0.02	47
INEDVb	0.59	0.59	1	0.10	0.18	47
INTDVb	0.67	0.67	1	0.37	0.09	47
INCRb	0.60	0.62	1	0.10	0.19	47
IBOLRb	2.07	2.12	2.35	1.57	0.20	47

*1. CNIFa, INEDVa, INTDVa, INCRa, IBOLRa denote new infection rate, inner-day volatility of stock index, inter-day volatility of the stock index, increase rate of the stock index, and the inter-bank overnight lending rate in 2020, respectively; while CNIFa, INEDVa, INTDVa, INCRa, IBOLRa denote the variables in 2021*.

*2. Variables of INEDVa, INTDVa, INCRa, INEDVb, INTDVb, INCRb are featured scaled*.

On account of these time-series data's possible stationary or unstationary nature, first, we apply a unit root test. In the unit root test, both the Augmented Dickey-Fuller (ADF)test and the Phillips-perron (PP) test are examined to mutually confirm the results. Specifically, we employ the Schwarz Information Citeria (SIC) and check all the possible cases including “constant term”, “constant + trend”, and “none” to judge the variables' optimal lags. The testing results are contained in Table 2. INCRa, INEDVa, INTDVa, CNIRa, and IBOLRa signify the increase rate of the stock index, the inner-day volatility of the stock index, the inter-day of the stock index, the new infected rate per million people and inter-bank overnight lending rate in 2020, while INCRb, INEDVb, INTDVb, CNIRb, and IBOLRb are the corresponding variables in 2021. The unit root test results that all the dependent variables such as INCRa and INCRb, INEDVa, and INEDVb, INTDVa, and INTDVb are integrated at level, denoted as I(0), while both the independent variables of CNIRA and CNIRB are integrated in order 1, denoted as I(1).

**Table 2:** Results of unite root tests.

**Variable**	**Type**	**ADF**		**PP**	
		**Level**	**1st dif**.	**Level**	**1st dif**.
**Year 2020**					
LnCNIRa	Intercept	−0.94 [0.77]	−7.15*** [0.00]	−1.28 [0.63]	−10.66 [0.00]
	Trend and intercept	−1.50 [0.82]	−5.49*** [0.00]	−3.02 [0.14]	−10.50 [0.00]
	None	0.79 [0.88]	−7.07*** [0.00]	0.58 [0.84]	−10.51 [0.00]
LnINCRa	Intercept	−6.92*** [0.00]	−11.31*** [0.00]	−6.94*** [0.00]	−49.90*** [0.00]
	Trend and intercept	−6.98*** [0.00]	−11.18*** [0.00]	−7.01*** [0.00]	−45.09*** [0.00]
	None	−2.04** [0.04]	−11.43*** [0.00]	−3.06*** [0.00]	−48.84*** [0.00]
LnINEDVa	Intercept	−4.99*** [0.00]	−8.61*** [0.00]	−4.94*** [0.00]	−17.58*** [0.00]
	Trend and intercept	−4.95*** [0.00]	−8.52*** [0.00]	−4.90*** [0.00]	−17.35*** [0.00]
	None	−1.98** [0.05]	−8.70*** [0.00]	−2.36** [0.05]	−17.94*** [0.00]
LnINTDVa	Intercept	−8.21*** [0.00]	−8.24*** [0.00]	−8.23*** [0.00]	−40.22*** [0.00]
	Trend and intercept	−8.23***[0.00]	−8.17*** [0.00]	−8.27*** [0.00]	−40.05*** [0.00]
	None	−1.40*** [0.15]	−8.33*** [0.00]	−2.18*** [0.03]	−40.17*** [0.00]
LnIBOLRa	Intercept	−4.29*** [0.00]	−6.63*** [0.00]	−3.92*** [0.00]	−10.93*** [0.00]
	Trend and intercept	−4.26*** [0.00]	−6.56*** [0.00]	−3.81** [0.02]	−10.68*** [0.00]
	None	−1.00 [0.28]	−6.70*** [0.00]	−0.56 [0.47]	−11.15*** [0.00]
**Year 2021**					
LnCNIRb	Intercept	−2.25 [0.19]	−9.54*** [0.00]	−2.20 [0.21]	−9.54*** [0.00]
	Trend and intercept	−2.86 [0.18]	−9.46*** [0.00]	−2.88 [0.18]	−9.46*** [0.00]
	None	−1.25 [0.19]	−9.63*** [0.00]	−1.10 [0.24]	−9.62*** [0.00]
LnINCRb	Intercept	−6.26*** [0.00]	−10.94*** [0.00]	−6.27*** [0.00]	−26.71*** [0.00]
	Trend and intercept	−6.39*** [0.00]	−5.91*** [0.00]	−6.38*** [0.00]	−26.16*** [0.00]
	None	−1.14 [0.23]	−11.07*** [0.00]	−1.54 [0.11]	−26.94*** [0.00]
LnINEDVb	Intercept	−5.53*** [0.00]	−9.50*** [0.00]	−5.51*** [0.00]	−20.74*** [0.00]
	Trend and intercept	−5.51*** [0.00]	−9.40*** [0.00]	−5.46*** [0.00]	−20.94*** [0.00]
	None	−2.49** [0.01]	−9.61*** [0.00]	−2.28** [0.02]	−21.00*** [0.00]
LnINTDVb	Intercept	−7.95*** [0.00]	−12.94*** [0.00]	−8.16*** [0.00]	−46.55*** [0.00]
	Trend and intercept	−7.87*** [0.00]	−12.80*** [0.00]	−8.19*** [0.00]	−46.54*** [0.00]
	None	−1.33 [0.17]	−13.09*** [0.00]	−1.98** [0.05]	−45.70*** [0.00]
LnIBOLRb	Intercept	−4.42*** [0.00]	−9.39*** [0.00]	−4.42*** [0.00]	−12.64*** [0.00]
	Trend and intercept	−4.35*** [0.00]	−9.31*** [0.00]	−4.35*** [0.00]	−12.61*** [0.00]
	None	−0.78 [0.37]	−9.47*** [0.00]	−0.78 [0.37]	−12.77*** [0.00]

1. CNIFa, INEDVa, INTDVa, INCRa, IBOLRa denote new infection rate, inner-day volatility of the stock index, inter-day volatility of the stock index, increase rate of the stock index, and the inter-bank overnight lending rate in 2020, respectively; while CNIFa, INEDVa, INTDVa, INCRa, IBOLRa denote the variables in 2021. 2. p-values are provided in brackets. 3. (***), (**), (*) significant at 1, 5, 10% levels, respectively.

Since both of the ADF and PP tests suggest that the orders of the variables between the dependent variables and independent variables are mixed of *I(0)* and *I(1)*, then we employ The ARDL approach in the estimation because the ARDL approach has advantages in dealing with co-integration, regardless of the integration orders and samples scale. It also considers the classification of dependent and independent variables and results in showing the relationships in both long and short-terms. Specifically, we follow the Pesaran et al. (36) approach, which examines the co-integration by estimating an unrestricted error correction model (UECM) based on an equation. UECM for ARDL bounds testing with two independent variables is as the following formulas:

$$\begin{array}{r} \Delta LnY_{t}=\alpha_{0\;}\;+\overset{n}{\underset{i=1}{\sum }}\alpha_{1i}\Delta LnY_{t-i}+\overset{n}{\underset{i=0}{\sum }}\alpha_{2i}\Delta LnCNIR_{t-i}\\ +\overset{n}{\underset{i=0}{\sum }}\alpha_{3i}\Delta LnIBOLR_{t-i}+\;\alpha_{4}Ln\;Y_{t-1\;}\;+\;\alpha_{5}Ln\;CNIR_{t-1\;}\;\\ +\;\alpha_{6}Ln\;IBOLR_{t-1\;}{\;+\;e}_{t}\;\left(a\right) \end{array}$$

Where, *Y* is stock price volatility, INCR, INEDV, INTDV are respectively estimated in Model A1, Model A2, Model A3 of 2020, and Model B1, Model B2, and Model B3 of 2021. α_0_ is constant coefficient, α_4_, α_5_, and α_6_ are long-run coefficients, α_1*i*_, α_2*i*_, and α_3*i*_ are short-run coefficients. *e*_*t*_ is an error term of white noise. Then the null hypothesis and the alternative are set based on the above equations, as is shown as follows:

$$\begin{array}{l} H_{0}:\;\alpha_{4}\;=\;\alpha_{5}\;=\alpha_{6}=0 \end{array}$$

$$\begin{array}{l} H_{1}:\;\alpha_{4}\;\neq \;\alpha_{5}\;\neq \alpha_{6}\neq 0 \end{array}$$

Where, *H*_0_ denotes that no relationship existed in the long run, otherwise *H*_1_ implies the existence of long-term association. In the ARDL bounds test, The Wald test is used to check the possible co-integration. If the resulting F-statistics is smaller than the corresponding lower critical bound, there is no co-integration. If the value of *F*-statistics is bigger than the corresponding upper critical bound, there exists co-integration. If the value is between the two, it is equivocality whether these series are co-integrated or not.

As long as a relationship of co-integration between independent variables and dependent variables are checked, the ARDL model of long-run and short-run relationship can be developed with equations as follows:

Long-term equation:

$$\begin{array}{r} LnY_{t}=\beta_{0\;}\;+\overset{n}{\underset{i=1}{\sum }}\beta_{1i}LnY_{t-i}+\overset{a}{\underset{i=1}{\sum }}\beta_{2i}LnCNIR_{t-i}\\ +\overset{b}{\underset{i=1}{\sum }}\beta_{3i}LnIBOLR_{t-i}\;+e_{t}\;\left(b\right) \end{array}$$

Short-term equation:

$$\begin{array}{r} \Delta LnY_{t}=\lambda_{0\;}\;+\overset{m}{\underset{i=1}{\sum }}\lambda_{1i}\Delta LnY_{t-i}+\overset{c}{\underset{i=1}{\sum }}\lambda_{2i}\Delta LnCNIR_{t-i}\\ +\overset{d}{\underset{i=1}{\sum }}\lambda_{3i}\Delta LnIBOLR_{t-i}\;+\lambda_{4}{\left(ECT\right)}_{t-1}\;+e_{t}\;\left(c\right) \end{array}$$

Where, *n* is the number of variables, m is the maximum of lags' number. *a, b, c*, and *d* are the lag lengths of the variables. In particular, the determinants of the lag length are based on the resulting value of Akaike information criteria (AIC) and Schwarz information criteria (SIC). *ECT* is the error correction term of the long-run equation. Thus, the ARDL approach based on Pesaran et al. (36) is established to estimate the long-run and short-run relationship between the variables.

## Empirical Results

The empirical results of the ARDL approach contain two sequences: Model A and Model B, which are the estimations of COVID-19 pandemic's impact on stock prices in 2020 and 2021, respectively. The results of optimal length adopted in The ARDL bounds test, recommended by AIC and SIC, is shown in Table 3. We develop six UECM models, Model A1, A2, and A3, INCRa, INEDVa, and INTDVa are employed as dependent variables, respectively, to detect possible impacts in 2020, while Model B1, B2, and B3, INCRb, INEDVb, and INTDVb are employed as dependent variables to detect possible impacts in 2021. In the models, IBOLRa and IBOLRb are introduced, respectively, as control variables. The optimal lags of each model recommended by Table 3 are used in the estimation of ARDL model's long-run and short-run equations. In models A1, the optimal lag length is recommended of 2 AIC and 1 SIC, we take the length lag of 1 by AIC. Similarly the optimal lag length in Model A2 and A3 is taken of 3 and 2 by SIC. In the sequences of 2021 data, in Model A1, A2, and A3 all of the optimal lag lengths are recommended of 1 by AIC and SIC.

**Table 3:** Optimal lags of models.

**Model**	**AIC**	**SIC**
**Year 2020**		
A1	0.96 (2)	1.77 (1)
A2	1.67 (3)	2.37 (1)
A3	2.57 (2)	1.23 (1)
**Year 2021**		
B1	−7.13 (1)	−6.63 (1)
B2	−6.55 (1)	−6.10 (1)
B3	−7.86 (1)	−7.36 (1)

*The optimal number of lags is provided in parentheses*.

Adopting the recommended lag length by AIC and SIC above, we employ the Wald test to check the possible integration. The results (contained in Table 4) show that the F-statistics in all the models are larger than corresponding upper bound values at a 1% significance level. It signifies that the null hypothesis of no co-integration in all the models is rejected. Hence, the ARDL approach is tailored to investigate the relationship of long-run and short-run, adopting the optimal lag length recommend in Table 3.

**Table 4:** Results of ARDL bounds test.

**Model**	**AIC**	**SIC**
**Year 2020**		
A1	22.53^***^	22.08^***^
A2	13.72^***^	13.72^***^
A3	25.06^***^	25.06^***^
**Year 2021**		
B1	14.20^***^	14.20^***^
B2	12.69^***^	12.69^***^
B3	19.54^***^	19.54^***^
**ARDL bounds test, critical value**		
**Significant**	**Critical value**	**Critical value**
**level**	**of upper bound**	**of lower bound**
1%	6.36	5.15
5%	4.85	3.79
10%	4.14	3.17

(***) *is significant at 1% significance level*.

The estimating results of long-run coefficients and short-run coefficients are demonstrated in Table 5. In the year of 2020, it shows that the coefficients of CNIRa are negative to INCRa in the short-run at 5% significance level, whereas no significant coefficient in the long-term is detected at a conventional significance level, regardless of model specifications. It implies that the COVID-19 epidemic negatively impacted China's stock market index only during a short time period. However, in the year 2021, the coefficient of CNIRb is significant at the conventional significance level and positively correlated to INCRb in the long-term, while in the short-term it is statistically insignificant. It means that, in the epilog of the COVID-19 breakout, the epidemic may have contributed to the increase of China's stock index. In addition, in Model B2, it shows a negative impact of CNIRb on INEDVb in the short-run, denoting that the epidemic reduced the stock's inner-day volatility and the impact lasted not very long. Likewise, IBOLR indicates a negative impact on stock index and inner-day volatility in model A1 and A2, and inner-day volatility in model B2. It implies that an increase in the inter-bank overnight lending rate reduced SSCI and mitigated inner-day volatility in 2020, whereas just significantly reduced inner-day volatility in 2021.

**Table 5:** Empirical results in long run and short run.

**Variables**	**Model A1**	**Model A2**	**Model A3**
**Year 2020**			
**Long-run coefficient**			
C	−0.68*** (−3.13)	−0.72*** (−2.66)	−0.36*** (−3.00)
CNIF	0.57 (0.35)	1.85 (0.92)	0.01 (0.01)
IBOLR	−0.17 (−0.55)	−0.23 (−0.60)	−0.22 (−0.13)
**Short-run coefficient**			
c	−0.01 (−0.23)	−0.00 (−0.08)	−0.03 (−1.14)
Δ CNIF	−5.17* (−1.64)	−3.67 (−1.06)	−0.18 (−0.10)
Δ IBOLR	−0.49** (−2.32)	−0.50** (−2.22)	−0.18 (−1.57)
**Variables**	**Model B1**	**Model B2**	**Model B3**
**Year 2021**			
**Long-run coefficient**			
C	−0.68** (−2.06)	−0.27 (−0.48)	−1.03*** (−3.58)
CNIF	1.78* (1.86)	2.68 (1.60)	0.39 (0.58)
IBOLR	−0.10 (−0.23)	−0.83 (−1.11)	0.73* (1.92)
**Short-run coefficient**			
c	0.00 (0.02)	−0.01	0.00 (0.09)
Δ CNIF	1.24 (0.62)	−7.57** (−2.59)	−2.33 (−1.43)
Δ IBOLR	0.01 (0.04)	−1.19** (−2.60)	0.71*** (2.85)

*1. CNIFa, INEDVa, INTDVa, INCRa, IBOLRa denote new infection rates, inner-day volatility of the stock index, inter-day volatility of the stock index, increase rate of the stock index, and the inter-bank overnight lending rate in 2020, respectively; while CNIFa, INEDVa, INTDVa, INCRa, IBOLRa denote variables in 2021*.

*2. t-values are provided in parentheses*.

3. (***), (**), (*) significant at 1, 5, 10% levels, respectively.

The results revealed by Model B1 and B2 are different from the previous investigations and even economic common sense, in which the direct impacts of the epidemic on stock are negative (2, 3, 38, 39) or increase its fluctuation (4, 5, 28). However, considering the epidemic's influence on government and central bank policies tailoring, this phenomenon can be interpreted. Responses to policies intervention in the stock market have been suggested by a growing number of literature [e.g., (56–59)]. As for fiscal policy, most of the papers find positive influences of government expenditure and negative influences of taxes on stock prices [e.g., (60, 61)]. Even the government budget balance is acknowledged to be one of the main factors impacting the economic growth and stability that affect stock market returns (62) and thereby its price. As for monetary policy, studies on conventional monetary intervention (such as a change in policy interest rates) and unconventional monetary intervention (such as a change in monetary easing and liquidity support) have established its relationship with regard to stock price and its volatility (56, 58, 62–65). Specifically, as highlighted by Rogalski and Vinso (66), money supply has a lagged positive impact on stock return. With a similar suggestion about the direct impact of liquidity injection on the stock market, Cecioni et al. (67) further denotes that funding conditions of the policies may also influence prices through mitigating the friction of the finance system. He also addressed that the “portfolio channel” is one of two transmission channels by which monetary policy influences the economy. Moreover, the effects of monetary policy on stock prices can also be impacted by factors such as investors' trust (68) and expectations (67). In particular, some investigations provide evidences that the effect of policy information (65) and policy announcements (63) on stocks are significant. In the initial year of the COVID-19 outbreak, China's government and central bank had implemented unprecedented strong expansionary fiscal^2^ and monetary^3^ policies to counter the epidemic's shock on the economy. Under the logic that these stimulation policies may gradually fade out and return to “normal” as the epidemic slows down, the phenomenon may be due to the fact that in the post COVID-19 epidemic era, stock investors are more concerned about the impact of policies' withdraws implemented during the outbreak of the epidemic, than the epidemic itself.

## Conclusions and Policy Implications

The main objective of the study is to explore the long-run and short-run impact of the COVID-19 epidemic on China's daily stock price volatility during and after its outbreak. As *I(0)* and *I(1)* variables are mixed, the ARDL bounds test approach is employed to examine the existence of co-integration and the relationships of the long-run and short-run between daily new COVID-19 infections and China's market volatility. It was found that the COVID-19 epidemic decreased China's stock index and rise exacerbate its volatility during the period of COVID-19's outbreak (2020), as is suggested in Baiget al. (69), Bai et al. (2), Rahman et al. (3), and Dai et al. (27), although the impact was in the short-term. However, in the period of post COVID-19's outbreak (2021), the epidemic has a positive long-run impact on the stock prices and a negative short-run impact on its inner-day volatility. A plausible explanation is that in the post-epidemic era, the infections stabilize investors' expectations for the maintenance of the existing stimulating policies, which were introduced to fight against the epidemic's negative impact on the economy, thereby increasing the stock price and mitigating the inner-day volatility, rather than decreasing its price and exacerbating its fluctuation in the pandemic's initial year. This study extends the existing literature of COVID-19's impact on stock price volatility, which mainly focuses on the price itself and the period of the epidemic's outbreak. It also provides empirical evidence with regard to the epidemic's different impacts on China's stock market in the period when the pandemic was roughly controlled and gradually slowed down.

From a policy perspective, the study's outcome to a certain extent helps to respond to the causation of the complicated volatility of stock prices during different periods of the COVID-19 epidemic and provide insights on effective policy intervention in the forthcoming period. First, the government and central bank should prepare a policy cupboard such as liquidity injection and interest rate adjustment to counter possible fluctuations in the stock market. Second, the exits of the stimulus fiscal and monetary policies countering the pandemic's shock ought to take the influence factors of the stock market into consideration. A gradual and orderly way out of the stimulus policies can stabilize investors' expectations and trust, reduce the objectionable influence of the policies' transition and the risk of debt shock. In particular, it is imperative to provide a special grace period for the most severely affected sectors during the pandemic (e.g., travel, hotel, and restaurant sectors), and small and medium enterprises (SME) of which recovery is slower and more sensitive to debt risk, with policies such as preferential interest rates, targeted crediting, and easing. Moreover, the negative association between IBOLR and inner-day volatility unveiled by the research provides a news possible tool of intervention in stock price's inner-day fluctuations. That is, the monetary policy makers can manipulate the inter-bank overnight lending rate to reduce the epidemic's impact on the inner-day volatility of stock prices, instead of the traditional interest rate intervention as addressed in the most previous studies [e.g., (3, 41, 43, 45)], which may bring about significant and complicated influences on other economic variables (70) except stock prices. In addition, since the impact of COVID-19 on the stock market is no longer negative, an integrated policy is imperious to balancing social and economic sustainability, such as trade opening, fostering a favorable business environment, and supporting S&M enterprises, more than just focusing on public health, to achieve SDG.

Several issues remain for future study. First, it is possible to detect the impact discrepancy on stock prices and the volatility of different industry sectors and provide more precise and tailored policy implications. Second, investigate the possible asymmetric effect of the epidemic by employing an asymmetric estimation approach. Third, try to explore mediating or moderating variables to help find empirical evidence of the transmission mechanism from epidemic's impact to stock volatility which is the limitation of this study. Fourth, it is possible to explore whether other developing or emerging countries' stock markets have experienced similar phenomenon in the post-epidemic period.

## Data Availability Statement

The raw data supporting the conclusions of this article will be made available by the authors, without undue reservation.

## Author Contributions

CJ performed the material preparation, data collection, and analysis, wrote the first draft of the manuscript, commented on previous versions of the manuscript, contributed to the study conception and design, read, and approved the final manuscript.

## Funding

The authors thanks the financial support of Philosophy & Social Science Fund of Shaoxing City, China (145138).

## Conflict of Interest

The author declares that the research was conducted in the absence of any commercial or financial relationships that could be construed as a potential conflict of interest.

## Publisher's Note

All claims expressed in this article are solely those of the authors and do not necessarily represent those of their affiliated organizations, or those of the publisher, the editors and the reviewers. Any product that may be evaluated in this article, or claim that may be made by its manufacturer, is not guaranteed or endorsed by the publisher.

## Footnotes

^1^The total market value of China is 11.92 trillions RMB in October, 2021, equals 75.19% of GDP in 2020.

^2^In 2020, China expanded its government budget deficit by 1 trillion RMB, special bonds by 1.6 trillion RMB, and increased public expenditure by 0.81 trillion RMB, reduced taxes by 2.5 trillion RMB (Collected from government website of the People Republic of China: https://www.gov.cn/).

^3^In 2020, China's center bank has reduced the deposit reserve ratio to 9.4% which is the lowest in 5 years, lowered the benchmark interest rate twice to 2.95%, and put in a total of 1.75 trillion RMB base money to provide long-term liquidity to the market (Collected from the central bank website of the People's Republic of China: http://www.pbc.gov.cn/).
